# Cause of death for patients with breast cancer: discordance between death certificates and medical files, and impact on survival estimates

**DOI:** 10.1186/s13690-021-00637-w

**Published:** 2021-06-23

**Authors:** Hava Izci, Tim Tambuyzer, Jessica Vandeven, Jérôme Xicluna, Hans Wildiers, Kevin Punie, Nynke Willers, Eva Oldenburger, Els Van Nieuwenhuysen, Patrick Berteloot, Ann Smeets, Ines Nevelsteen, Anne Deblander, Harlinde De Schutter, Patrick Neven, Geert Silversmit, Freija Verdoodt

**Affiliations:** 1 Department of Oncology, KU Leuven - University of Leuven, Herestraat 49 box 7003-06, B-3000 Leuven, Belgium; 2 Belgian Cancer Registry, Research Department, Brussels, Belgium; 3 Department of General Medical Oncology, University Hospitals Leuven, B-3000 Leuven, Belgium; 4 Department of Gynecological Oncology, University Hospitals Leuven, B-3000 Leuven, Belgium; 5 Department of Radiation Oncology, University Hospitals Leuven, B-3000 Leuven, Belgium; 6 Department of Gynecology and Obstetrics, University Hospitals Leuven, B-3000 Leuven, Belgium; 7 Department of Surgical Oncology, University Hospitals Leuven, B-3000 Leuven, Belgium

**Keywords:** Breast cancer, Cause of death, Death certificates, Misclassification, Cause-specific survival, Relative survival

## Abstract

**Background:**

Registration and coding of cause of death is prone to error since determining the exact underlying condition leading directly to death is challenging. In this study, causes of death from the death certificates were compared to patients’ medical files interpreted by experts at University Hospitals Leuven (UHL), to assess concordance between sources and its impact on cancer survival assessment.

**Methods:**

Breast cancer patients treated at UHL (2009–2014) (follow-up until December 31st 2016) were included in this study. Cause of death was obtained from death certificates and expert-reviewed medical files at UHL. Agreement was calculated using Cohen’s kappa coefficient. Cause-specific survival (CSS) was calculated using the Kaplan-Meier method and the relative survival probability (RS) using the Ederer II and Pohar Perme method.

**Results:**

A total of 2862 patients, of whom 354 died, were included. We found an agreement of 84.7% (kappa-value of 0.69 (95% C.I.: 0.62–0.77)) between death certificates and medical files. Death certificates had 10.7% false positive and 4.5% false negative rates. However, five-year CSS and RS measures were comparable for both sources.

**Conclusion:**

For breast cancer patients included in our study, fair agreement of cause of death was seen between death certificates and medical files with similar CSS and RS estimations.

## Background

Accurate information on the principal cause of death, resulting in reliable cause-specific survival estimates, could be important for dependable estimation of disease-specific mortality, support treatment decision making and the allocation of health care resources.

In Belgium, the principal or underlying cause of death is derived from the death certificate, which is completed by a physician and captured in a coding system i.e., the Tenth Revision of the International Classification of Diseases (ICD-10), by registrars [1]. Accuracy of the principal cause of death in death certificates compared to a second, verified clinical source has been investigated in other countries, for patients with breast cancer [2–6] and other cancers [7–16]. Due to discordant reporting of cause of death, some of these studies recommend improved reporting of cause of death classification [17–20]. Since breast cancer has a favorable prognosis compared to some other cancers types, reporting the cause of death may be more difficult, particularly in patients with comorbidities. In Belgium, an in-depth review of the principal causes of death with an investigation of different survival measures has not been done yet.

Both relative survival (RS) and cause-specific survival (CSS) are approaches applied to estimate net survival of cancer patients. Cancer registries often use RS, as it does not need cause of death information, which is not always available at the population level. Both methods have weaknesses that could introduce bias in the net survival estimation. CSS requires accurate cause of death information from death certificates, whereas RS requires a disease-free reference group. Some studies have recommended the use of RS over CSS in breast cancer patients, because it is less susceptible to misclassification errors [21–27]. In this study, we compare the principal cause of death between death certificates and expert-reviewed medical files in a cohort of breast cancer patients at University Hospitals Leuven (UHL), a large-scale tertiary hospital with a specialized breast center in Belgium. Additionally, we explore its impact on CSS, and compare this with relative survival-based approaches to estimate net survival.

## Methods

### Study population

All female patients with a first invasive breast cancer diagnosis between January 1st, 2009 and December 31st, 2014, and treated in UHL were included in this study. Tumor and patient characteristics such as age, year of diagnosis, histological grade and tumor stage (TNM classification system, 6th and 7th edition) were obtained at the Belgian Cancer Registry (BCR) [28].

### Data sources

In order to calculate and compare the cause-specific survival (CSS) and relative survival (RS), different data sources were used. The vital status of patients, available up to December 31st, 2016, was obtained from linkage with the Belgian Crossroads Bank for Social Security (CBSS) using the patients’ social security number [29]. Patients with unknown vital status or deceased patients with missing cause of death information were excluded from analyses. For CSS, cause of death information was extracted from two sources: the death certificates obtained from the regional authorities in Flanders, Brussels and Wallonia, and the medical files obtained from UHL. For RS, the population life tables from Statistics Belgium were used [30].

When someone dies, cause of death and associated conditions are described in the death certificate by a certified physician. These death certificates are collected physically and electronically by regional authorities: the ‘Agentschap Zorg en Gezondheid’ for Flanders [31], ‘Observatoire de la Santé et du Social de Bruxelles-Capitale’ for Brussels [32], and ‘Agence pour une Vie de Qualité’ for Wallonia [33]. International coding and classification rules are applied to the certificates in accordance with ICD-10 [1]. Principal cause of death is derived from the chain of events that resulted in death. Coding principal cause of death is automatically done for a subset (40%) of death certificates using international coding software (IRIS software) [34, 35], while 49% percent are coded semi-automatically, and 11% are manually reviewed by an encoder based on the wording or phrasing of the physician to determine principal cause of death [31, 36].

The death certificate is designed to state the chain of events leading to death, thus, the immediate, intermediate, underlying and associated cause of death. The immediate cause is the cause that has led directly to the passing of the patient, which can be caused by or coincide with the intermediate and underlying cause of death. The associated causes of death are important contributing factors to the death. For instance, if a patient with breast cancer develops brain metastases, but dies from a brain hemorrhage, the immediate cause of death would be the hemorrhage, the intermediate cause the brain metastases and the underlying cause breast cancer. The latter would be defined as the principal cause of death. A possible associated cause of death could be, for example, pre-existing atherosclerosis of blood vessels in the brain.

Besides death certificates, information from medical files can be a second source of information about cause of death that can be used to calculate CSS. Information from expert-reviewed medical files was considered the gold standard in this study. A physician (P.N.) from UHL checked cause of death information twice for all deceased breast cancer patients from the available medical files. In case of a discordant or unknown cause of death, the case was flagged and an expert panel consisting of 7 physicians from UHL (H.W., K.P., N.W., E.O., E.V.N., P.B. and A.D.) performed a blinded verification of principal cause of death. If there was a discordance between the two, another member of the expert panel randomly performed a second blinded verification to select the principal cause of death. Finally, the principal cause of death was determined by majority decision.

All experts from the panel individually consulted the electronic medical files of patients that were assigned to them. When necessary, the experts additionally followed-up with the physician of the last patient contact, and consulted E-health (i.e., a platform for protected health information exchange among health care providers) [37]. The principal cause of death was defined as ‘breast cancer’, if this disease initiated the chain of events leading up to death and if the patient did not have an accident or injury that resulted in death. Breast cancer can initiate the chain of events leading up to the death in case of presence of breast cancer metastases, irrespective of the survival period of the patient. Death by the treatment of breast cancer is not considered as death due to breast cancer.

### Definition of survival measures

Different survival measures were calculated, i.e., CSS and RS [38, 39]. CSS only considers deaths due to breast cancer as an event, with cause of death being obtained from either death certificates or medical files. RS is defined as the ratio of overall survival (with death of any cause as an event) from the breast cancer patient cohort and expected survival of a comparable cohort from the general population, matched on sex, age, diagnosis year and region. Net survival, which encompasses the survival that would be observed if the only possible cause of death was the cancer under study, can be estimated with CSS or RS-based approaches. Survival time for patients was calculated from the incidence date to date of death or until last known date alive. Follow-up in death certificates was available up to end of 2016.

### Statistical analyses

Agreement between principal cause of death from death certificates and medical files was investigated by calculating the Cohen’s Kappa coefficient (κ-value) [40]. Concordance was investigated further by correlating κ-value with tumor and patient characteristics as age, diagnosis year, histological grade, tumor stage (combined pathological and clinical stage, TNM classification system) and tumor multiplicity. For all tumor and patient characteristics, subgroups were created. The κ-value was calculated for every subgroup separately. The Spearman’s correlation coefficient (ρ) was then calculated to measure the association strength between the subgroup and κ-value (*p*-value cutoff at 0.05) [41]. Subgroups in which stage or grade were unknown were excluded from the subgroup calculation. All analyses were performed with SAS 9.4 (SAS Institute, Cary, NC, USA) within the SAS Enterprise Guide software (version 7.15 of the SAS System for Windows).

CSS was calculated based on the principal cause of death from death certificates and medical files. CSS considered the survival time from date of diagnosis until the date of death from breast cancer (outcome of interest), death due to other causes (censored) or until last known date alive (censored). CSS estimation were performed with the Kaplan-Meier method in SAS [42]. Next, RS was calculated and compared with CSS. RS was calculated by the Ederer II method in SAS and R (R Core Team, 2017) [43, 44], and the more recent Pohar Perme method in R [45]. The SAS code uses broad pre-specified time intervals in the actuarial approach (mostly 1-year broad intervals), whereas the R code uses data driven time intervals (at each event and censoring time).

## Results

A total of 2862 breast cancer patients of which 354 died and for which cause of death information was available in both data sources, were included in the analyses (Table 1). The median follow-up period was 54.6 months. Blinded review for principal cause of death was performed by the expert panel in 70 cases, of which 8 patients needed a second blind verification. Concordance in principal cause of death between both sources showed a 4.5% false negative proportion (*n =* 16), and 10.7% false positive proportion (*n =* 38) (Table 2). False negatives were patients who were misclassified as having died from another cause than breast cancer, and false positives were patients who were misclassified as having died from breast cancer. The κ-value was 0.69 (95% C.I.: 0.62–0.77) [46].

**Table 1:** General patient and tumor characteristics (*n* = 2,862)

Category	Subgroups	N of patients	(%)
**Age at time of diagnosis**	<40 years	188	6,6
	40-49	562	19,6
	50-59	778	27,2
	60-69	709	24,8
	70-79	423	14,8
	≥80	202	7,1
**Year of diagnosis**	2009	489	17,1
	2010	448	15,7
	2011	468	16,4
	2012	483	16,9
	2013	500	17,5
	2014	474	16,6
**Stage at diagnosis**	Stage 0	47	1,6
	Stage I	1063	37,1
	Stage II	1174	41,0
	Stage III	357	12,5
	Stage IV	158	5,5
	Stage unknown	63	2,2
**Tumor Grade**	Grade 1	319	11,1
	Grade 2	1378	48,1
	Grade 3	1126	39,3
	Grade unknown	39	1,4
**Multiple tumors**	No	2733	95,5
	2+	129	4,5
**Treatment**^**a**^	Breast Conserving Surgery	1374	48,0
	Mastectomy	1311	45,8
	Radiotherapy	2354	82,3
	Chemotherapy	1296	45,3
	Hormonal therapy	2344	81,9
**Morphology**	Ductal	2118	74,0
	Lobular	337	11,8
	Other	407	14,2

^a^Some patients receive multiple different treatment interventions within each subgroup (surgery or systemic therapy) during their follow-up period

**Table 2:** Concordance/discordance table for the principle cause of death between medical files reviewed by board of experts (gold standard), and death certificates (*n* = 354 deaths)

		Medical files		Total
		Other Causes	Breast cancer	
Death certificates	Other causes	136 (38.4%)	16 (4.5%)	152 (42.9%)
	Breast cancer	38 (10.7%)	164 (46.3%)	202 (57.1%)
Total		174 (49.2%)	180 (50.9%)	354 (100%)

For false negatives, the most common cause of death in death certificates was primary cancer at the site of metastasis instead of breast cancer (*n* = 6 or 37.5%). None of the false negatives reported breast cancer in the listed immediate, intermediate, underlying or associated causes of death in the death certificate. Three out of 16 false negatives (18.8%) reported the ICD-10 code for ill-defined and unknown cause of death (ICD-10 code R99.0) as the principal cause of death. Other causes of misclassification were registration of another disease, another primary cancer or comorbidities from the patients’ history reported as principal cause of death (*n* = 7 or 43.7%). Seventeen out of 38 false positives, had their principal cause of death from medical files (i.e., not breast cancer) listed as intermediate or immediate cause of death in their death certificates. Some of these patients died from an acute unrelated death (stroke or cardiac arrest), that was reported as death from breast cancer (*n = 6)* in the death certificate.

Next, κ-value was calculated according to subgroups (Table 3). The agreement of principal cause of death between both sources had a weak inverse correlation with increasing age, stage and diagnosis year (n.s., *p* > 0.05). The Spearman’s correlation coefficients were − 0.7, − 0.8 and − 0.26 for increasing age, stage and diagnosis year respectively, thus correlation was lower in older age subgroups, higher stage and patients with a more recent year of diagnosis, however the *p*-value was not significant (n.s., *p* > 0.05). The agreement was classified as ‘fair’ in the subgroup with stage IV at diagnosis [47].

**Table 3:** Agreement analyses (kappa statistic) for different patient subgroups (grouped based on patient and tumor characteristics), comparing principal cause of death from death certificates and medical files

Category	Subgroups	Number of patients alive	Number of deceased patients (FU 31-12-2016)	Kappa statistic	Spearman’s correlation coefficient and *p*-value
Age	<40 years	177	11	ND^a^	Rho= -0.7 *P*-value= 0.23
	40-49	525	37	0.64	
	50-59	736	42	0.83	
	60-69	649	60	0.73	
	70-79	334	89	0.54	
	≥80	87	115	0.50	
Year of diagnosis	2009	393	96	0.77	Rho= -0.26 *P*-value= 0.67
	2010	379	69	0.59	
	2011	398	70	0.71	
	2012	429	54	0.67	
	2013	472	28	0.78	
	2014	437	37	0.54	
Stage at diagnosis	Stage 0	46	1	ND^a^	Rho= -0.8 *P*-value= 0.33
	Stage I	1019	44	0.76	
	Stage II	1053	121	0.69	
	Stage III	288	69	0.75	
	Stage IV	56	102	0.35	
	Stage unknown^b^	46	17	0.43	
Grade of tumor	Grade 1	301	18	ND^a^	ND^c^
	Grade 2	1223	155	0.58	
	Grade 3	960	166	0.81	
	Grade unknown^b^	24	15	0.86	
Multiple tumors	Yes	94	35	0.66	ND^c^
	No	2414	319	0.69	

^a^Not defined due to events within subgroup being too small

^b^ subgroups with unknown stage or grade were not included in Spearman analyses

^c^not defined due to subgroup without ranked variables

To investigate the impact of misclassification of cause of death on survival measurements, 5-year CSS was calculated based on both sources separately. CSS calculated from principal cause of death obtained from medical files resulted in slightly higher 5-year CSS estimates (93.1% (95% C.I.: 91.9–94.1)), compared to principal cause of death obtained from death certificates (92.3% (95% C.I.: 91.2–93.4)) (Table 4).

**Table 4:** 5-year cause-specific survival (CSS) using primary cause of death information from medical files and death certificates (Follow-up until December 31st, 2016)

	5-year breast cancer specific survival, % (95% CI)		
	Inclusion of known COD from medical files and death certificates (354 deaths)	Inclusion of known COD from death certificates (373 deaths)	Inclusion of known COD from medical files (402 deaths)
CSS (death certificates)	92.3 (91.2, 93.4) (Events^a^: 183/2862)	92.7 (91.6, 93.7) (Events^a^: 188/2882)	
CSS (medical files)	93.1 (91.9, 94.1) (Events^a^: 162/2862)		93.1 (91.9, 94.1) (Events^a^: 165/2726)

^a^Events are breast cancer-related deaths within 5 years after diagnosis

Finally, different net survival approaches were used in order to compare these estimates (Table 5). A small difference could be seen in survival estimates from RS calculated with Pohar Perme and Ederer II method and CSS as calculated with the Kaplan-Meier method.

**Table 5:** 5-year net survival estimates (relative survival (RS) and cause-specific survival (CSS)) using different methods^a^

	5-year net survival, % (95% CI)
RS - Pohar Perme (in R)	93.2 (91.5, 94.8)
RS - Ederer II (in R)	93.7 (92.3, 95.2)
RS - Ederer II (in SAS)	92.8 (91.3, 94.2)
CSS (Medical files) (in SAS)	93.1 (91.9, 94.1)
CSS (Death certificates) (in SAS)	92.3 (91.2, 93.4)

^a^Patients with COD available in both medical files and death certificates were selected (354 deaths). Years of diagnosis 2009-2014, follow-up of vital status up to December 31st, 2016

## Discussion

This study evaluated accuracy of death certificates by validation of causes of death against a medical file review by a board of experts. Additionally, we investigated the impact of misclassification of cause of death on CSS. We found fair agreement between causes of death reported in death certificates and medical files, although this kappa-value interpretation has been defined slightly differently in publications over the years and should be interpreted relative to the setting [46–49]. Further, CSS with cause of death information obtained from medical files was slightly higher as a result of less deaths due to breast cancer, compared to survival using causes of death from death certificates, but was generally similar. Expert review was useful to identify and solve difficult cases where cause of death was unclear or difficult to determine.

First, we investigated discordant causes of death between death certificates and medical files. Among the false negatives (4.5% of cases), misattribution of breast cancer-specific death in death certificates was linked to the presence of comorbidities, metastases (from the primary breast cancer), or unspecified causes. We found more false positives (10.7% of cases) or over-reporting of breast cancer deaths than underreporting. Our results are consistent with literature for breast cancer that state more false positive cases of breast cancer-related deaths in comparison to false negatives [4, 5], although earlier studies from 1980s reported underestimation of breast cancer as principal cause of death in death certificates [2, 3].

Subsequently, we looked into trends of misclassification in specific subgroups based on patient and tumor characteristics. For age, diagnosis year and stage at diagnosis, Spearman’s correlation coefficient could be calculated as a measure for the strength of relationship between the agreement factor and subgroups. Although not statistically significant, a weak inverse correlation was seen for age, stage and diagnosis year. A previous study in Geneva, Switzerland by Schaffar et al. [5] found more misclassification in older adults and patients with advanced disease. Older patients with cancer are more likely to have multiple comorbidities, which could lead to an increased risk of misclassifying the principal cause of death. Besides that, patients with metastases at diagnosis are more likely to have misclassification of cause of death since their site of metastases might be reported as the primary cause of death.

Several studies have investigated and validated the reporting and misclassification of causes of death in breast cancer patients, since the quality of death certification has been questioned [2–6]. Previous studies obtained discordance rates of 8.8% [5], 9.0% [2, 3] and 10.0% [6]. Our study showed a discordance rate of 15.2% between death certificates and expert-reviewed medical files, which was higher than previous studies. Coding of cause of death in death certificates according to international ICD-10 guidelines is semi-automatic, which helps to unify all codes with rule definitions, but details of the chain of causes of death can get lost in this coding system [36]. In addition, certification errors by the clinician responsible for assigning the causes of death in the death certificate, for example due to incomplete information, could lead to misclassification, as the IRIS system is dependent on the quality and information mentioned on the death certificates.

Identifying cause of death in medical files could be difficult in breast cancer patients with comorbidities, as it may be unclear if the patient has died from cancer, comorbidities or complications related to the cancer treatment. Breast cancer in particular is less lethal than some other cancers or comorbidities. This makes it more challenging to identify the cause of death correctly, since patients are more likely to die from non-cancer related causes. Other cancers with more lethal outcome, such as lung cancer, have shown higher overestimation of death due to cancer than breast cancers [16].

We validated death certificates by using an expert board that actively checked different data sources to evaluate medical history of the patient and designate an accurate principal cause of death. Review of medical files is routinely done for all patients in the Geneva cancer registry [5], since it is useful to have exact registration of causes of death for patients and obtain exact cause of death information. These specialized registrars are trained to carry out yearly follow-up of the registry with the aim to calculate CSS with this cause of death information. Unfortunately, a manual review of medical files is often not possible in the real-life setting, given labor intensity and costs. Guidelines for registrars and physicians have been developed according to ICD-10 in order to improve reliability of cause of death reporting. Periodic reviews of (a sample of) cause of death data and implementation of these guidelines would be beneficial in the future, as this could help to have more accurate disease-specific survival data and respond to epidemiological trends. When limited resources are available, such reviews could be restricted to patients with more discordance, such as patients with older age and higher disease stage.

Consequently, we wanted to see what the impact of misclassification of cause of death would be on survival. The survival results from these approaches were very similar. For breast cancer patients included in the study, RS measure that does not require cause of death information was comparable to CSS measures. A recent publication by Wissing et al. [17] recommends reporting and interpreting the CSS, RS and overall survival measure altogether to complement each other. Detailed description of the procedure and data sources to identify cause of death when reporting these measures is also recommended in the future.

The limitations of this study were that for a few cases, cause of death information was not available in any of the available medical files and could not be investigated further by consulting external sources. We did, however, have the chance to use extensive medical files from UHL, a large-scale tertiary Hospital in Belgium with a specialized breast center. This allowed strict adherence to guidelines and adequate clinical follow-up for patients. It would also be interesting to investigate the classification rate for causes of death in breast cancer patients in a secondary hospital in the future, to compare these results.

## Conclusions

For patients with breast cancer, we observed a fair agreement of cause of death classification between death certificates and verified medical files in UHL. Attribution of cause of death to comorbidities was the most common reason for discordant reporting of breast cancer-specific death. CSS calculated with cause of death information from death certificates following ICD-10 rules showed similar CSS compared to medical files. Results for CSS and RS were similar, as well. Although there are clear guidelines for registration of cause of death, periodic reviews of the implementation of these rules and continuous training of registrars and physicians may be needed in order to obtain accurate cause of death data, and measure survival based on these data. Registries should ideally combine information from different sources and review discordant cases.

## Data Availability

The data that support the findings of this study are available upon reasonable request. The data can be given within the secured environment of the Belgian Cancer Registry, according to its regulations, and only upon approval by the Information Security Committee.

## Abbreviations

BCR: Belgian Cancer Registry
CBSS: Crossroads Bank for Social Security
CSS: Cause-Specific Survival
ICD-10: Tenth Revision of the International Classification of Diseases
RS: Relative Survival
UHL: University Hospitals Leuven
